# *Notes from the Field*: Investigation of Patients Testing Positive for Yellow Fever Viral RNA After Vaccination During a Mass Yellow Fever Vaccination Campaign — Angola, 2016

**DOI:** 10.15585/mmwr.mm6610a5

**Published:** 2017-03-17

**Authors:** Andrew T. Boyd, Diambi Dombaxe, Rosa Moreira, M.S. Oliveira, Eusebio Manuel, Carlos Navarro Colorado, Tatiana M. Lanzieri

**Affiliations:** ^1^Epidemic Intelligence Service, CDC; ^2^Division of Global Health Protection, Center for Global Health, CDC; ^3^Angola Field Epidemiology and Laboratory Training Program, Ministry of Health, Republic of Angola; ^4^Ministry of Health, Republic of Angola; ^5^Division of Viral Diseases, National Center for Immunization and Respiratory Diseases, CDC.

The yellow fever outbreak declared in Angola in January 2016 soon became the largest recorded yellow fever outbreak in the country’s history. In response, the Angola Ministry of Health, supported by the World Health Organization (WHO), conducted mass yellow fever vaccination campaigns beginning in February 2016 for all persons aged ≥6 months. By June 2016, a total of 11.6 million yellow fever vaccine doses had been distributed among a national population of 25 million. Because of the urgency of distributing vaccine to stop the outbreak, surveillance for cases of yellow fever after vaccination and serious adverse events after immunization (AEFIs) was not implemented. However, CDC and the Angola Field Epidemiology and Laboratory Training Program conducted an investigation of patients with a history of yellow fever vaccination and symptoms of yellow fever disease whose specimens tested positive for yellow fever viral RNA by reverse transcription–polymerase chain reaction (RT-PCR) to assess whether such cases could represent vaccine failure or AEFIs.

Although no yellow fever vaccine efficacy studies have been conducted, the vaccine is reliably immunogenic; worldwide, only five postvaccine yellow fever cases have been described (*1*). Neutralizing antibodies develop by day 10 after vaccination in 80% of yellow fever vaccinees (*1*). Primary yellow fever vaccine recipients have self-limited, vaccine-derived, physiologic viremia, typically during days 3–4, although this postvaccination viremia can last as long as 2 weeks. Thus, the detection of yellow fever viral RNA by RT-PCR testing before postvaccination day 3 or after day 13 could represent wild-type infection (acquired either before vaccination or later if there is vaccine failure) or yellow fever vaccine-associated viscerotropic disease (YEL-AVD), a rare but serious AEFI in which the vaccine-derived virus proliferates in multiple organs after primary vaccination. The symptoms of YEL-AVD are similar to those of naturally acquired yellow fever, typically beginning by postvaccination day 10; vaccine-derived viremia can persist beyond day 13. The risk for YEL-AVD is 0.3–0.4 cases per 100,000 yellow fever vaccine doses distributed among U.S. travelers; however, risk estimates in the context of mass vaccination campaigns in Africa are limited (*2*). Therefore, symptom onset within 10 days after vaccination and viremia on or after day 3 could represent YEL-AVD, physiologic viremia, or yellow fever after vaccine failure.

National epidemiologic and linked laboratory data, including RT-PCR results, were reviewed to identify all suspected yellow fever cases (defined in the outbreak as the occurrence of fever and jaundice) in persons who also had a history of yellow fever vaccination and who received a positive RT-PCR test result during January 1–May 11, 2016. Vaccination was recorded in the database based on self-report or presentation of a WHO vaccination card. Database records of yellow fever vaccination among patients who received positive RT-PCR test results were confirmed through review of original suspected yellow fever case surveillance forms and patient medical records and through telephone interviews with patients or their families. The intervals from vaccination date to symptom onset date and from vaccination date to sample collection date were calculated. The uniformity of distribution of vaccination date was assessed using Chi-square goodness-of-fit testing.

Among 2,907 suspected cases of yellow fever, 459 (16%) patients had documentation of receipt of yellow fever vaccine. Among these, 376 (82%) also had documented RT-PCR results, including 51 (14%) who received positive RT-PCR test results. Among these 51 patients, 50 had surveillance forms, and seven had medical records for review; 20 patients or their families could be contacted to confirm vaccination. Among the 51 patients who received positive RT-PCR test results, symptom onset occurred after vaccination in 32 (63%). Among the remaining 19, five were excluded because they had not been vaccinated, eight because their symptoms preceded vaccination, and six because they had no documented vaccination date.

Among the 32 patients who received positive RT-PCR test results after vaccination, 24 (75%) were male, the mean age was 20 years (standard deviation = 12 years), and 13 (41%) died. Eighteen (56%) received positive test results for yellow fever viral RNA after postvaccination day 13, and 11 (34%) received positive test results during days 0–13; the sample collection date was missing for three patients. Symptom onset occurred during postvaccination days 0–10 in 17 (53%) patients, and after day 10 in 15 (47%). Distribution of vaccination dates was uniform, implying no clustering by date. Information about location of vaccination was not available to assess clustering by place.

Insufficient clinical and laboratory information was available to determine which of the 32 patients who received positive RT-PCR test results had wild-type infection (either before vaccination or as a result of vaccine failure) or physiologic viremia after vaccination. A lack of supplementary information also precluded determining whether any of these 32 patients met diagnostic criteria for YEL-AVD (*2*). Although nucleotide sequencing can distinguish wild-type from vaccine-derived yellow fever viremia, and viral RNA quantification can aid in the diagnosis of YEL-AVD, additional testing on specimens from five of these patients performed at a reference laboratory found no detectable viral RNA, thus precluding viral RNA sequencing.

After this investigation, the Angola Ministry of Health modified the suspected yellow fever case surveillance form to include the location of vaccination and instructions to send specimens from patients who develop symptoms and receive positive RT-PCR test results after vaccination for specialized testing. In addition, personnel from the Angola Ministry of Health and WHO investigate such cases, gathering comprehensive clinical and laboratory data, to improve surveillance for both yellow fever after vaccination and serious AEFIs.
